# TEMPORAL RELATIONSHIPS OF PERSON- AND TASK-CENTERED DEMENTIA CARE AND MEALTIME BEHAVIORS: SEQUENTIAL ANALYSIS

**DOI:** 10.1093/geroni/igac059.533

**Published:** 2022-12-20

**Authors:** Wen Liu, Yelena Perkhounkova, Maria Hein, Roger Bakeman

**Affiliations:** University of Iowa, Iowa City, Iowa, United States; University of Iowa, Iowa City, Iowa, United States; University of Iowa College of Nursing, Iowa City, Iowa, United States; Georgia State University Department of Psychology, Atlanta, Georgia, United States

## Abstract

Person-centered mealtime care is highly recommended in dementia care. While current research examined associative relationships between person- and task-centered care and resident mealtime behaviors, few studies evaluated their temporal associations. Videotaped mealtime observations (N=160) involving 36 staff and 27 residents (53 staff-resident dyads) in 9 nursing homes were coded. Staff person-centered and task-centered approaches were conceptualized as antecedents of resident positive behaviors, functional impairments, and resistive behaviors using 5-, 10-, and 15-second time windows. Immediately after staff person-centered approaches, resident positive and resistive behaviors were more likely (p range=<.001–.29) and functional impairments less likely (p range=<.001–.62) with diminished effects in time. Immediately after staff task-centered approaches, resident positive behaviors were less likely (p range=<.001–.09). Person-centered mealtime care should be individualized, context-based, and resident-oriented. Resident resistiveness to care may be behavioral responses to person-centered care indicating mismatch to individual preferences and needs, warranting adequate awareness and appropriate assessment.

